# Shame‐related disorders in patients with atopic dermatitis and psoriasis – An exploratory, cross‐sectional interview study on the prevalence and correlates of body dysmorphic disorder and social anxiety disorder

**DOI:** 10.1111/ddg.15892

**Published:** 2025-10-16

**Authors:** Clara Wülfing, Carsten Spitzer, Laura Lübke, Steffen Emmert, Alexander Thiem

**Affiliations:** ^1^ Clinic and Policlinic for Psychosomatic Medicine and Psychotherapy University Medical Center Rostock Rostock Germany; ^2^ Clinic and Policlinic for Dermatology and Venereology University Medical Center Rostock Rostock Germany

**Keywords:** Atopic dermatitis, body dysmorphic disorder, cross‐sectional study, psoriasis, shame‐related disorders, social anxiety disorder

## Abstract

**Background and objectives:**

Atopic dermatitis (AD) and psoriasis (Pso) are frequently associated with psychological distress. This study evaluated the prevalence and correlates of shame‐related disorders (SRD), namely body dysmorphic disorder (BDD) and social anxiety disorder (SAD), in patients with AD and Pso.

**Patients and methods:**

A monocentric, cross‐sectional study was conducted in adult patients from 07/2023 to 03/2024. A trained clinical psychologist assessed subjects for BDD and SAD. Additionally, subjects completed the DLQI. Objective severity of their disease was physician‐rated using the EASI or PASI.

**Results:**

One hundred and fifty‐one patients were included, n = 55 (36.4%) with AD and n = 96 (63.6%) with Pso. Among all study participants, the point and lifetime prevalence of SRD was 17.2% and 31.8%, respectively. Point and lifetime prevalence for BDD was 10.6% and 26.5%, and for SAD 12.6% and 17.2%. There were no differences in the point or lifetime prevalence of BDD or SAD between patients with AD or Pso. SRD were associated with younger age and female sex. DLQI was significantly reduced in those suffering from SRD.

**Conclusions:**

Our results indicate that SRD are prevalent in AD and Pso and should therefore be further investigated for their use in routine clinical practice.

## INTRODUCTION

Atopic dermatitis (AD) and psoriasis (Pso) represent the most common chronic inflammatory skin diseases affecting approximately between 2% (Pso) and 4.5% (AD) of the adult general population worldwide.^1^, ^2^ Due to their chronic, often relapsing, and remitting course, both conditions cause considerable disease burden including intense itch, sleep disturbances, impaired psychosocial functioning, reduced quality of life (QoL), and poor mental health.^3^, ^4^, ^5^, ^6^ In addition, many patients with AD or Pso suffer from stigmatization and discrimination.^7^, ^8^ All these factors may interact negatively resulting in cumulative impairment over the life course,^9^ as well as in psychological distress and negative affectivity including depression, anxiety, and shame.

Not surprisingly, meta‐analytic evidence indicates significant associations of adult AD with depression and anxiety.^10^, ^11^, ^12^ For example, the odds of being clinically depressed were more than threefold higher among 162 patients with AD from 13 European countries compared to a control group.^13^ Longitudinal studies suggest that adult patients with AD have an elevated risk of developing major depression or other depressive disorders.^14^ Moreover, there is meta‐analytic evidence for an association between AD severity and depression.^10^ Similarly, clinically relevant anxiety and anxiety disorders are prevalent in adult AD.^13^, ^15^

Likewise, systematic reviews as well as meta‐analyses clearly show that adult Pso is related to depression and anxiety.^15^, ^16^, ^17^, ^18^ However, the prevalence of depression among adult patients with Pso varies considerably because of variations in study design, particularly depression assessment.^18^ Approaches using clinician‐administered diagnostic tools typically report lower prevalence rates of depression than those applying self‐report measures.^18^ A very similar picture arises concerning anxiety in Pso in that prevalence estimates are higher when assessed via self‐report than diagnostic interviews.^15^, ^17^ Pso severity has been found to be associated with increases in both depression^16^ and anxiety.^15^, ^17^

In contrast to depression and anxiety, shame is just beginning to attract scientific interest in dermatology.^19^, ^20^ This is particularly true for body dysmorphic disorder (BDD) and social anxiety disorder (SAD), which are considered shame‐related disorders (SRD).^21^, ^22^ BDD is characterized by an impairing and persistent preoccupation with a minor or perceived flaw in physical appearance; any minor abnormalities are overly exaggerated, and the level of preoccupation is clearly disproportionate resulting in significant distress as well as impairments in QoL and psychosocial functioning.^23^ Although research on BDD is increasing in dermatology,^24^, ^25^ there is little systematic knowledge on the prevalence and correlates of BDD in patients with AD or Pso. A most recent observational, cross‐sectional multicenter study among 5,487 consecutive dermatological outpatients in 17 European countries found that adult patients with AD or Pso had a more than sevenfold increased chance of self‐reported BDD symptoms compared with healthy skin controls; the estimated prevalence of BDD was 15.9% among AD patients and 13.9% among those with Pso.^26^ In dermatological outpatients in general, BDD was significantly related to younger age, female sex, and reduced QoL.^24^, ^26^

The main feature of SAD is an excessive fear of social or performance situations, in which embarrassment or humiliation is anticipated leading to avoidance behavior and significant impairments in various life domains.^27^ Findings regarding SAD in adults affected by Pso are rather inconsistent as indicated by a recent meta‐analysis.^28^ Although studies have found a high prevalence of SAD, there is a wide heterogeneity and risk of publication bias which is reflected by divergent prevalence rates according to the method of assessment ranging from 3% in studies using interviews to 42% in studies with self‐administered questionnaires.^28^ Of note, despite extensive research on anxiety in AD,^13^, ^15^ the prevalence of SAD is unknown. In sum, research on shame‐related disorders (i.e. BDD and SAD) among adult patients with AD and Pso is limited, inconclusive, and may be undermined by assessing mental health with self‐report measures susceptible to several kinds of bias.

In light of these considerations and findings, our explorative study aimed to address two research questions: *(1)* What is the point and lifetime prevalence of BDD and SAD in adult patients with AD or Pso, when semi‐structured interviews are applied? *(2)* How do lifetime diagnoses of BDD and SAD relate to age, gender, duration and severity of the respective skin disease, health‐related QoL, and current treatment?

## PATIENTS AND METHODS

### Study design and procedure

Data were collected during dermatology consultations at the outpatient department of the Clinic for Dermatology and Venereology, University Medical Centre Rostock (Germany). To be eligible, patients had to meet the following inclusion criteria: age ≥ 18 years, dermatologist‐confirmed diagnosis of either AD or Pso vulgaris, no cognitive impairment, and sufficient German language skills for responding to interview questions. Patients were excluded from the study if they had solely pustular, palmarplantar, guttate, or nail Pso. All eligible subjects were invited to participate in this study; after full explanation of the procedure and having given written informed consent, subjects were interviewed by the same trained clinical psychologist (CW). Afterwards, participants completed the *Dermatology Life Quality Index* (DLQI; see below). Demographic data were collected by patient self‐report. The severity and location of the skin disease were documented by the attending dermatologist using the *Psoriasis Area and Severity Index* (PASI)^29^, ^30^ or the *Eczema Area and Severity Index* (EASI).^31^ This cross‐sectional, observational study was approved by the local Institutional Review Board (approval number A 2023‐0051) and conformed to the principles of the Declaration of Helsinki. It was conducted between July 2023 and March 2024. Of 217 eligible patients (148 with Pso and 69 with AD), 163 individuals (75.1%) provided informed consent to participate. Another 12 patients had to be excluded because of missing data or withdrawal of their consent, resulting in a net study sample of 151 patients including 96 subjects with Pso and 55 with AD.

### Diagnostic interviews and self‐report measures

The assessment of shame‐related disorders followed a two‐stage procedure. At the beginning of the interview, body dysmorphic concerns and social anxieties were assessed using the respective screening questions from the clinical version of the *Structured Clinical Interview for DSM‐5* (SCID‐5‐CV):^32^ “Have you ever been very concerned that there was something wrong with your physical appearance or the way one or more parts of your body looked?” and “Have you ever been particularly nervous or anxious in social situations, for example during conversations or when meeting unfamiliar people?” In case the answer was positive, the following instruments were administered to further evaluate whether the diagnostic criteria for BDD and SAD had been met at any time in life (lifetime prevalence) as well as in the week prior to the assessment (point prevalence). SRD was defined as presence of either BDD or SAD, both lifetime and currently.

#### Yale‐Brown Obsessive‐Compulsive Scale Modified for Body Dysmorphic Disorder (BDD‐YBOCS)

BDD symptoms were further assessed using the German version of the BDD‐YBOCS,^33^, ^34^, ^35^ a 12‐item semi‐structured clinician‐administered interview. The BDD‐YBOCS assesses preoccupation with a perceived defect, compulsive behaviors, avoidance, and insight. The clinician rates each item on a scale from 0 (no symptoms) to 4 (extreme symptoms); thus, the scoring scale can span from 0 to 48, with a designated cut point of 20 or higher indicating the presence of BDD.^34^, ^36^ The psychometric properties of the BDD‐YBOCS were reported to be good to excellent.^34^, ^37^ For this study, not only the usual time frame (i.e. the past week) was referred to, but also the entire lifespan.

#### The Liebowitz Social Anxiety Scale (LSAS)

The LSAS (Liebowitz, 1987; German version by von Consbruch et al.^38^) is a clinician‐administered, semi‐structured interview for the assessment of SAD‐related symptoms. It uses two subscales that address social interaction (11 items) and performance (13 items), measuring an individual's fear and avoidance of social situations. Answers are given on a four‐point Likert scale, and each item is separately rated for fear and avoidance. The total score can range between 0 to 144 points, and a cut‐off score exceeding 30 effectively distinguishes individuals with and without social anxiety disorder.^39^ Prior research demonstrated robust psychometric properties for the LSAS.^40^ Again, lifetime and point prevalence (i.e. presence in the last week) were explored.

#### Dermatology Life Quality Index (DLQI)

The DLQI is a well‐established, generic self‐report measure of health‐related QoL in the last week among patients with skin diseases.^41^ Its ten items are rated on four‐point Likert scales generating scores between 0 and 30 with higher scores indicating a lower QoL. Reliability and validity have been established in numerous studies.^42^

### Data analysis

In addition to descriptive statistics, several between‐group comparisons between patients with AD and Pso with regard to shame‐related disorders were performed. For categorical variables, the χ^2^‐test was used. For continuous variables (e.g. DLQI data), the non‐parametric Mann‐Whitney U‐test was applied to account for small and divergent case numbers in the groups to be compared. Significance level was set at α < 0.05. All analyses were done with the *Statistical Package for the Social Sciences* (SPSS, version 27).

## RESULTS

### Study population

The study population comprised 65 women (43.0%) and 86 men (57.0%) with a mean age of 45.2 years (SD = 16.2; range: 18–85 years). Of those, 55 (36.4%) suffered from AD and 96 (63.6%) from Pso. A more detailed account of the sociodemographic and clinical characteristics including current treatment of the study population and the two subsamples is presented in Table 1.

**TABLE 1:** Sociodemographic and clinical characteristics of the study population.

	Study sample (n = 151)	AD patients (n = 55)	Pso patients (n = 96)
***Age*** *(years; M ± SD; range)*	45.2 ± 16.2 (18–85)	40.2 ± 16.6 (18–80)	48.0 ± 15.3 (19–85)
***Sex***			
Women	65 (43.0%)	26 (47.3%)	39 (40.6%)
Men	86 (57.0%)	29 (52.7%)	57 (59.4%)
***Marital status***			
Single, no partner	56 (37.1%)	26 (47.3%)	30 (31.3%)
Married/ steady partner	78 (51.7%)	20 (36.4%)	58 (60.4%)
Divorced/ separated	17 (11.3%)	9 (16.4%)	8 (8.3%)
***Education***			
High school	88 (58.3%)	28 (50.9%)	60 (62.5%)
Secondary school	53 (35.1%)	23 (41.8%)	30 (31.3%)
Other	10 (6.6%)	4 (7.3%)	6 (6.3%)
***Disease duration*** (years; M ± SD; range)	23.0 ± 16.7 (0–64)	27.3 ± 16.4 (0–64)	20.6 ± 16.4 (0–58)
***Visible lesion*** (yes)	94 (63.1%)	40 (75.5%)	54 (56.3%)
***Disease severity***			
EASI (M ± SD)		5.74 ± 7.59	–
PASI (M ± SD)		–	3.33 ± 6.02
***DLQ*I** (M ± SD)	5.05 ± 6.21	6.51 ± 6.88	4.21 ± 5.65
***Current treatment***			
No treatment	5 (3.3%)	1 (1.8%)	4 (4.2%)
Only topicals	24 (15.9%)	10 (18.2%)	14 (14.6%)
Only biologics	55 (36.4%)	11 (20.0%)	44 (45.8%)
Only others	14 (9.3%)	2 (3.6%)	12 (12.5%)
Topicals and others	15 (9.9%)	6 (10.9%)	9 (9.4%)
Biologics and topicals	34 (22.5%)	23 (41.8%)	11 (11.5%)
Biologics and others	2 (1.3%)	1 (1.8%)	1 (1.0%)
Biologics, topicals and others	2 (1.3%)	1 (1.8%)	1 (1.0%)

*Abbr*.: AD, atopic dermatitis; DLQI, Dermatology Life Quality Index; EASI, Eczema Area Severity Index; M, mean; PASI, Psoriasis Area Severity Index; Pso, psoriasis; SD, standard deviation; SRD, shame‐related disorders

Topicals include topically applied anti‐inflammatory drugs like steroids as monotherapy or combined with Vitamin D analogues. Others comprises oral drugs, e.g. JAK inhibitors, and UV phototherapy.

### Prevalence of BDD and SAD

**FIGURE 1:**
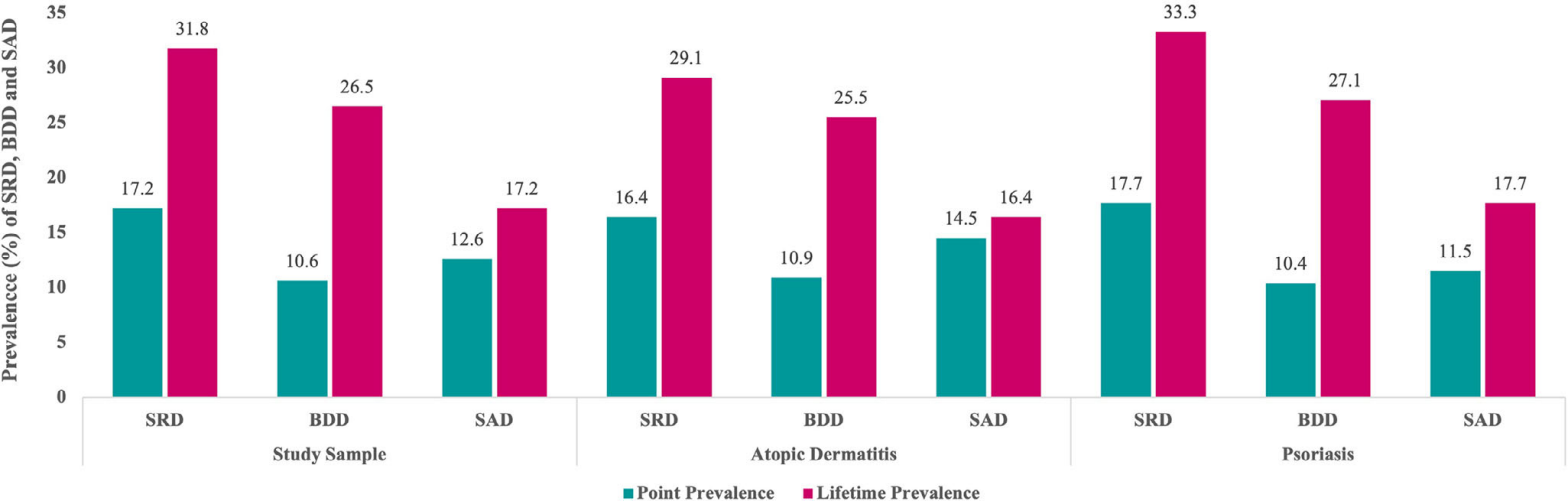
Comparison of point and lifetime prevalence rates of shame‐related disorders (SRD), body dysmorphic disorder (BDD), and social anxiety disorder (SAD) among patients with the chronic inflammatory skin diseases atopic dermatitis (AD) or psoriasis (Pso). There were no significant differences in point or lifetime prevalence between patients with AD and Pso.

Among all study participants with chronic inflammatory skin diseases, the point and lifetime prevalence of shame‐related disorders was 17.2% and 31.8%, respectively. The prevalence rates for BDD and SAD in patients with Pso and AD are given in Figure 1. Of note, there were no differences in the point or lifetime prevalence of BDD or SAD between patients with AD or Pso.

### Sociodemographic and clinical correlates of shame‐related disorders

Looking at the entire study sample, a lifetime diagnosis of any of the two shame‐related disorders (SRD) was associated with younger age and female sex; the same was true in patients with AD or Pso (Table 2). Interestingly, neither disease duration nor the visibility of skin lesions were related to the presence of SRD. Similarly, in either patients with AD or those with Pso, there was no association between skin disease severity (as measured with the EASI or PASI, respectively) and SRD. However, QoL was significantly lower in those currently suffering from an SRD, both in the entire study population as well as in both subsamples of patients with either AD or Pso. Given that Item 2 in the DLQI pertains explicitly to skin‐related shame (“Over the last week, how embarrassed or self conscious have you been because of your skin?”), we additionally investigated whether SRD might be associated to DLQI scores when excluding this item. We found nearly identical correlations for Qol scores both with (ρ = 0.320, p < 0.001) and without Item 2 (ρ = 0.323, p < 0.001). This was consistent for correlations with BDD and SAD as well (data not shown).

**TABLE 2:** Comparison of age, sex, disease severity and duration, and quality of life in patients with chronic inflammatory skin diseases with and without shame‐related disorders (SRD; lifetime).

	SRD +	SRD –	Statistics	
	*Total study sample*		*U/ χ^2^*	*p*
Age (years, M ± SD)	38.0 ± 15.9	48.5 ± 15.3	1,532.0	*< 0.001*
Women	30 (62.5%)	35 (34.0%)	10.862	*< 0.001*
Visible lesion	31 (64.6%)	63 (62.4%)	0.068	0.794
Disease duration (years; M ± SD)	20.0 ± 15.2	24.4 ± 17.2	2026.0	0.152
DLQI	6.64 ± 6.93	4.33 ± 5.75	1804.0	*0.012*
	***Atopic Dermatitis***			
Age (years, M ± SD)	30.9 ± 11.4	44.0 ± 17.0	160.0	*0.005*
Women	11 (68.8%)	15 (38.5%)	4.176	*0.041*
EASI (M ± SD)	5.64 ± 9.66	5.78 ± 6.71	273.0	0.469
Visible lesion	11 (68.8%)	29 (78.4%)	0.559	0.455
Disease duration (years; M ± SD)	25.7 ± 14.6	28.0 ± 14.6	261.5	0.504
DLQI	9.25 ± 9.27	5.38 ± 5.38	253.0	.272
	***Psoriasis***			
Age (years, M ± SD)	41.6 ± 16.8	51.3 ± 13.5	672.5	*0.006*
Women	19 (59.4%)	20 (31.3%)	6.996	*0.008*
PASI (M ± SD)	3.97 ± 6.92	3.01 ± 5.55	897.5	0.316
Visible lesion	20 (62.5%)	34 (53.1%)	0.762	0.383
Disease duration (years; M ± SD)	17.1 ± 14.9	22.4 ± 17.0	821.5	0.176
DLQI	5.29 ± 5.01	3.69 ± 5.91	677.5	*0.011*

*Abbr*.: DLQI, Dermatology Life Quality Index; EASI, Eczema Area Severity Index; M, mean; PASI, Psoriasis Area Severity Index; SD, standard deviation; SRD, shame‐related disorders

Focusing specifically on the lifetime diagnoses of BDD and SAD, almost the same pattern of findings emerged (Tables 3, 4): In the entire study population and among patients with AD and Pso, both BDD and SAD were linked to younger age and female sex, but not to disease severity, duration or visibility of skin lesions. The only exception was that among AD patients, the gender distribution did not differ between those with SAD compared to those without SAD (Table 4). Interestingly, findings regarding QoL were inconsistent. While DLQI scores were higher in those with BDD or SAD, respectively, the only significant differences was observed between AD patients with vs. without SAD.

**TABLE 3:** Comparison of age, sex, disease severity and duration, and quality of life in patients with chronic inflammatory skin diseases with and without body dysmorphic disorder (BDD; lifetime).

	BDD +	BDD –	Statistics	
	*Total study sample*		*U/ χ^2^*	*p*
Age (years, M ± SD)	37.4 ± 16.5	48.0 ± 15.2	1,366.0	*< 0.001*
Women	27 (67.5%)	38 (34.2%)	13.272	*< 0.001*
Visible lesion	24 (60.0%)	70 (64.2%)	0.224	0.636
Disease duration (years; M ± SD)	22.4 ± 15.1	23.3 ± 17.3	2107.0	0.936
DLQI	6.13 ± 7.02	4.67 ± 5.89	1835.5	0.154
	***Atopic Dermatitis***			
Age (years, M ± SD)	30.9 ± 12.0	43.3 ± 16.9	148.0	*0.007*
Women	11 (78.6%)	15 (36.6%)	7.381	*0.007*
EASI (M ± SD)	5.14 ± 9.86	5.94 ± 6.78	230.0	0.270
Visible lesion	9 (64.3%)	31 (79.5%)	1.286	0.257
Disease duration (years; M ± SD)	27.7 ± 13.8	27.2 ± 17.3	269.5	0.944
DLQI	8.14 ± 9.28	5.95 ± 5.89	270.0	.741
	***Psoriasis***			
Age (years, M ± SD)	40.9 ± 17.6	50.7 ± 13.5	605.0	*0.012*
Women	16 (61.5%)	23 (32.9%)	6.465	*0.011*
PASI (M ± SD)	3.95 ± 7.31	3.10 ± 5.51	831.0	0.507
Visible lesion	15 (57.7%)	39 (55.7%)	0.030	0.862
Disease duration (years; M ± SD)	19.4 ± 15.1	21.1 ± 17.0	852.5	0.849
DLQI	5.00 ± 5.27	3.93 ± 5.80	691.0	.113

*Abbr*.: DLQI, Dermatology Life Quality Index; EASI, Eczema Area Severity Index; M, mean; PASI, Psoriasis Area Severity Index; SD, standard deviation

**TABLE 4:** Comparison of age, sex, disease severity and duration, and quality of life in patients with chronic inflammatory skin diseases with and without social anxiety disorder (SAD; lifetime).

	SAD +	SAD –	Statistics	
	*Total study sample*		*U/ χ^2^*	*p*
Age (years, M ± SD)	35.9 ± 14.0	47.1 ± 16.0	977.0	*< 0.001*
Women	17 (65.4%)	48 (38.4%)	6.393	*0.011*
Visible lesion	17 (65.4%)	77 (62.6%)	0.071	0.789
Disease duration (years; M ± SD)	17.2 ± 14.5	24.3 ± 16.9	1204.0	0.054
DLQI	7.15 ± 7.65	4.11 ± 5.81	1241.5	0.063
	***Atopic Dermatitis***			
Age (years, M ± SD)	30.1 ± 11.9	42.1 ± 16.8	112.0	*0.030*
Women	5 (55.6%)	21 (45.7%)	0.296	0.586
EASI (M ± SD)	8.77 ± 12.19	5.14 ± 6.37	173.0	0.439
Visible lesion	8 (88.9%)	32 (69.6%)	1.417	0.234
Disease duration (years; M ± SD)	23.7 ± 15.8	28.1 ± 16.5	157.5	0.343
DLQI	12.78 ± 10.10	5.28 ± 5.41	116.5	*0.039*
	***Psoriasis***			
Age (years, M ± SD)	38.9 ± 14.4	50.0 ± 14.8	401.0	*0.009*
Women	12 (70.6%)	27 (34.2%)	7.689	*0.006*
PASI (M ± SD)	3.48 ± 6.63	3.29 ± 5.93	589.0	0.420
Visible lesion	9 (52.9%)	45 (57.0%)	0.092	0.762
Disease duration (years; M ± SD)	13.8 ± 12.9	22.1 ± 16.8	476.5	0.070
DLQI	4.18 ± 3.63	4.21 ± 5.66	551.0	.270

*Abbr*.: DLQI, Dermatology Life Quality Index; EASI, Eczema Area Severity Index; M, mean; PASI, Psoriasis Area Severity Index; SD, standard deviation

Considering the relatively low severity of the skin diseases, the analyses were repeated after stratifying the study population into a subsample with mild skin disease severity (EASI ≤ 15 or PASI ≤ 10) and one with moderate‐to‐severe degree (EASI > 15 or PASI > 10). The findings are given in the online supplementary Tables S1–S3. For the subsample with a mild severity, basically the same patterns emerged, i.e. shame‐related disorders were related to younger age and female sex, but not to disease duration, severity, or visibility of the skin lesions; the association with QoL remained inconsistent. Of note, the subsamples with moderate‐to‐severe degree were too small to perform statistical analysis (6 patients in the AD group, and 9 patients in the PSO population). However, looking at the tables, descriptive analyses revealed a very similar pattern.

Because the current treatments of the study population proved to be very heterogeneous, the analyses of its possible link with lifetime prevalence rates of SRD were limited to two comparisons: *(1)* patients with and without biologics, and *(2)* patients with biologics vs. those being treated exclusively with topicals. These comparisons were chosen due to their prevalence in the sample, as they reflected the most commonly used treatment approaches. The online supplementary Table S4 presents the detailed results. In short, while patients treated with biologics had numerically higher lifetime prevalence rates for SRD, BDD, and SAD than those without biologics or participants only on topical therapies, these differences were not statistically significant.

## DISCUSSION

Drawing on prior research highlighting the relevance of shame in dermatological patients, this exploratory, cross‐sectional study investigated the prevalence of the shame‐related disorders BDD and SAD in patients with AD and Pso. To the best of our knowledge, this is the first study using a semi‐structured interview to assess BDD, which is considered the gold standard. In contrast, most studies on BDD in dermatological patients have relied exclusively on self‐report measures.^24^, ^25^ The few approaches with clinician‐administered interviews^43^ included dermatological patients in general but did not report the frequency of BDD in patients with AD or Pso. Additionally, this is the first study to determine the prevalence of SAD in patients with AD, which has not been investigated before.

The lifetime prevalence of BDD among those with chronic inflammatory skin disease was 26.5%, without significant differences between patients with AD and Pso. These figures are almost twice as high as estimates reported by a recent study ranging between 13.9% for Pso and 15.9% for AD,^26^ which are slightly above the point prevalence found in our study. These striking differences might be attributed to divergent methods for the diagnosis of BDD: While Schut and colleagues^26^ applied a self‐reported screening measure asking whether the participant has ever suffered from typical BDD symptoms, we used a semi‐structured, clinician‐administered interview. Using the same interview, another study reported a BDD prevalence of 36% among dermatological patients in general,^43^ which is threefold higher than the 10.5% of clinically relevant BDD symptoms found in 5487 patients with common dermatological conditions in the study of Schut et al.^26^ Thus, it might be concluded that besides the type of survey (self‐reported vs. clinician‐administered), the screening instrument and a variety of other response biases might influence the true prevalence rates of BDD.^44^

Regarding SAD, its point and lifetime prevalence in patients with chronic inflammatory skin disease were 12.6% and 17.2%, respectively; again, there were no significant differences between patients with AD and Pso. As this is the first study on the prevalence of SAD in adult patients affected by AD based on a clinician‐administered interview, our findings cannot be related to figures from prior research. However, in patients with a lifetime diagnosis of AD, the point prevalence of any anxiety disorder was 8.5%.^45^ Additionally, generalized anxiety disorder (GAD) was found to be associated with AD, but prevalence rates were not reported.^46^

In contrast to AD, meta‐analytic evidence indicates a high prevalence of SAD in patients with Pso, which has been averaged to 15% across all studies.^28^ This figure is quite similar to the rates found in our sample of Pso patients. However, looking at studies employing interviews to diagnose SAD, the pooled prevalence was only 3%,^28^ which is not only much lower than the findings in our investigation but also below the prevalence of SAD in the general population.^27^ These inconsistencies are most likely caused by methodological artifacts as the above‐mentioned studies used predominantly clinical interviews or the Mini International Neuropsychiatric Interview (MINI) performing poorly in detecting SAD.^47^, ^48^

In line with prior research^16^, ^24^, ^26^ our findings indicate that SRD in patients with chronic inflammatory skin diseases was associated with younger age and female sex. As expected and consistent with other studies,^24^, ^26^ current shame‐related disorders in general as well as BDD and SAD in particular were found to negatively impact QoL. Neither the severity nor the duration of the underlying skin disease was related to the lifetime prevalence of any shame‐related disorder. This finding came somewhat surprising because such a link might be assumed, particularly when considering that increases in both depression and anxiety have been reported to be associated with the severity of AD^10^ and Pso.^15^, ^16^, ^17^ Possibly, the study was underpowered to detect small to medium effects because the number of participants was too low, particularly in the subsample of patients with AD. Another explanation and the main caveat of our study refers to the study populations's relatively low skin disease activity,^49^, ^50^ which might be attributed to the success of previous and current therapies or to a selection bias. However, stratifying the sample into mild vs. moderate‐to‐severe disease activity did not result in any different associations. Of note, we did not detect a link between visible lesions and SRD, BDD or SAD. One might speculate that the liability to develop BDD or SAD is independent of possibly shame‐inducing factors, which is supported by prior findings.^26^, ^43^ Further studies are warranted to shed light on this issue.

Although our study has several strengths (for example, a thorough diagnostic process including a semi‐structured interview for both BDD and SAD conducted by the same trained clinical psychologist), certain methodological limitations warrant discussion. First, as this was a monocentric study, the number of participants was rather small. Second, the heterogeneity of the current treatment regimes did not allow any clear‐cut analyses, which makes it impossible to draw any firm conclusions on the relation between therapy and the presence of SRD on the basis of our data. Third, recruiting participants from the outpatient department of a university clinic may limit external validity; it remains unclear whether the findings can be generalized to other settings. Fourth, although QoL was significantly lower in subjects with SRD, absolute DLQI values were only above the cut‐off of 10 in SRD‐positive AD patients. Finally, the lack of an age‐ and sex‐matched control group of skin‐healthy individuals might be considered as a limitation.

Notwithstanding these caveats, our study as well as prior findings^26^, ^28^, ^43^ indicate that the shame‐related disorders BDD and SAD are prevalent in dermatological patients with AD and Pso. Pending replication and considering that shame‐related disorders impact QoL, future research will clarify whether their early detection and appropriate treatment are likely to alleviate patients’ distress and to improve their QoL.

## FUNDING

C.W. received a research grant from Almirall Hermal GmbH. S.E. is supported by the German Research Foundation (DFG: EM 68/13‐1; EM 68/15‐1; GRK 2901/1), the Federal Ministry of Education and Research (BMBF: 16GW0345), the European Union (HORIZON‐MSCA‐2022‐DN‐01, proposal number 101118430; PlasTHER COST Action CA20114), the Federal Ministry for Economic Affairs and Climate Action (BMWK: 03TN0019B), the Ministry of Economics, Infrastructure, Tourism and Labor of Mecklenburg‐West Pomerania (TBI‐V‐1‐349‐VBW‐120), and the European Regional Development Fund (ERDF: GSH‐20‐0054). The funders had no role in study design, data collection, analysis, or interpretation, writing of the manuscript, or the decision to submit the paper for publication.

## CONFLICT OF INTEREST STATEMENT

C.W. has received consultant's honoraria from Almirall. S.E. has received honoraria or travel support from Abbvie, Almirall, Amgen, Bristol‐Meyers Squibb, Cinogy, Galderma, Janssen, LEO, Malinckrodt, Mayne Genzyme Corporation, Merck Sharp & Dohme, Novartis, Oncobeta, Pierre‐Fabre, Pfizer, RheaCell, Sanofi, SolGel, SUN Pharma, Teion, and UCB. A.T. has received honoraria and/or travel support from AbbVie, Almirall, Boehringer Ingelheim, Bristol‐Meyers Squibb, Galderma, GlaxoSmithKline, Janssen, Kyowa Kirin, LEO, Lilly, Merck Sharp & Dohme, Novartis, Pfizer, Pierre Fabre, Recordati Rare Diseases, Sanofi, and UCB, and research support from Almirall and Cinogy. The other authors declare no conflicts of interest.

## Supporting information



Supplementary information
