# Supplementary material for: Shame‐related disorders in patients with atopic dermatitis and psoriasis – An exploratory, cross‐sectional interview study on the prevalence and correlates of body dysmorphic disorder and social anxiety disorder
Source: J Dtsch Dermatol Ges. 2025 Oct 16;24(2):196–204. doi: 10.1111/ddg.15892 (PMC12875151; doi:10.1111/ddg.15892)
Supplement: Supplementary file 1 — Supplementary information [file DDG-24-196-s001.docx]

TABLE S1 Comparison of age, sex, disease severity and duration, and quality of life in patients with chronic inflammatory skin diseases with and without shame-related disorders (SRD; lifetime), stratified according to disease severity (mild on the left side; moderate-to-severe on the right side)

|  | **Severity: Mild (n = 136)** | | | | **Severity: Moderte-to-severe (n = 15)** | |
| --- | --- | --- | --- | --- | --- | --- |
| ***Total study sample*** | ***SRD +*** | ***SRD –*** | ***Statistics*** | | ***SRD +*** | ***SRD –*** |
|  |  |  | *U/ χ^2^* | *p* |  |  |
| n (%) | 43 (31.6) | 93 (68.4) |  |  | 5 (33.3) | 10 (60.7) |
| Age (years; M ± SD) | 38.2 ± 16.3 | 48.8 ± 14.9 | 1239.5 | *< 0.001* | 36.6 ± 13.2 | 48.8 ± 19.3 |
| Women | 27 (62.8%) | 30 (32.3%) | 11.259 | *< 0.001* | 3 (60.0%) | 5 (50.0%) |
| Visible lesion | 26 (60.5%) | 54 (59.3%) | 0.015 | 0.901 | 5 (100.0%) | 9 (90.0%) |
| Disease duration (years; M ± SD) | 20.7 ± 15.4 | 24.8 ± 17.0 | 1679.5 | 0.187 | 13.0 ± 12.2 | 21.4 ± 19.8 |
| DLQI | 5.81 ± 6.28 | 3.83 ± 5.59 | 1461.5 | *0.011* | 15.50 ± 8.27 | 9.00 ± 5.31 |
| ***AD subsample*** |  |  |  |  |  |  |
| n (%) | 14 (28.6) | 35 (71.4) |  |  |  |  |
| Age (years; M ± SD) | 31.3 ± 11.7 | 43.2 ± 17.1 | 131.5 | *0.012* | 28.0 ± 12.7 | 51.0 ± 16.7 |
| Women | 10 (71.4%) | 13 (37.1%) | 4.720 | *0.030* | 1 (50.0%) | 2 (50.0%) |
| EASI (M ± SD) | 2.54 ± 2.60 | 4.07 ± 4.03 | 197.0 | 0.287 | 27.35 ± 15.34 | 20.75 ± 7.30 |
| Visible lesion | 5 (35.7%) | 7 (21.2%) | 1.087 | 0.297 | 2 (100.0%) | 3 (75.0%) |
| Disease duration (years; M ± SD) | 28.1 ± 13.5 | 29.1 ± 16.6 | 217.0 | 0.745 | 8.5 ± 12.0 | 19.0 ± 22.0 |
| DLQI | 7.36 ± 8.24 | 4.94 ± 5.05 | 216.0 | 0.518 | 22.50 ± 2.12 | 9.25 ± 7.46 |
| ***PSO subsample*** |  |  |  |  |  |  |
| n (%) | 29 (33.3) | 58 (66.7) |  |  |  |  |
| Age (years; M ± SD) | 41.5 ± 17.3 | 51.7 ± 12.5 | 543.5 | *0.007* | 42.3 ± 11.9 | 47.3 ± 22.2 |
| Women | 17 (58.6%) | 17 (29.3%) | 6.976 | *0.008* | 2 (66.7%) | 3 (50.0%) |
| PASI (M ± SD) | 2.05 ± 2.68 | 1.71 ± 2.57 | 720.5 | 0.266 | 22.50 ± 8.58 | 15.55 ± 10.21 |
| Visible lesion | 17 (58.6%) | 28 (48.3%) | 0.829 | 0.363 | 3 (100.0%) | 6 (100.0%) |
| Disease duration (years; M ± SD) | 17.1 ± 15.2 | 22.3 ± 16.8 | 691.0 | 0.176 | 17.5 ± 14.8 | 23.0 ± 20.2 |
| DLQI | 5.07 ± 5.09 | 3.16 ± 5.83 | 526.5 | *0.004* | 8.50 ± 2.12 | 8.83 ± 4.17 |

DLQI, dermatology life quality index; EASI, eczema area severity index; M, mean; PASI, psoriasis area severity index; SD standard deviation.

**TABLE S2** Comparison of age, sex, disease severity and duration, and quality of life in patients with chronic inflammatory skin diseases with and without body dysmorphic disorder (BDD; lifetime), stratified according to disease severity (mild on the left side; moderate-to-severe on the right side).

|  | **Severity: Mild (n = 136)** | | | |  | **Severity: Moderate-to-severe (n = 15)** | |  |
| --- | --- | --- | --- | --- | --- | --- | --- | --- |
| ***Total study sample*** | ***BDD +*** | ***BDD –*** | ***Statistics*** |  |  | ***BDD +*** | ***BDD –*** |  |
|  |  |  | *U/ χ^2^* | *p* |  |  |  |  |
| n (%) | 37 (27.2) | 99 (72.8) |  |  |  | 3 (20.0) | 12 (80.0) |  |
| Age (years; M ± SD) | 38.0 ± 16.8 | 47.9 ± 14.9 | 1162.5 | *< 0.001* |  | 30.0 ± 16.5 | 48.4 ± 17.9 |  |
| Women | 25 (67.6%) | 32 (32.3%) | 13.742 | *< 0.001* |  | 2 (66.7%) | 6 (50.0%) |  |
| Visible lesion | 21 (56.8%) | 59 (60.8%) | 0.184 | 0.668 |  | 3 (100.0%) | 11 (91.7%) |  |
| Disease duration (years; M ± SD) | 22.4 ± 15.4 | 23.9 ± 17.0 | 1724.5 | 0.727 |  | 22.5 ± 7.8 | 18.4 ± 19.3 |  |
| DLQI | 5.62 ± 6.55 | 4.02 ± 5.57 | 1473.5 | 0.076 |  | 15.50 ± 12.02 | 10.08 ± 5.92 |  |
| ***AD subsample*** |  |  |  |  |  | *n = 6* |  |  |
| n (%) | 13 (26.5) | 36 (73.5) |  |  |  | 1 (16.7) | 5 (83.3) |  |
| Age (years; M ± SD) | 31.7 ± 12.0 | 42.7 ± 17.1 | 136.0 | *0.026* |  | 19.0 | 48.2 ± 15.8 |  |
| Women | 10 (76.9%) | 13 (36.1%) | 6.387 | *0.011* |  | 1 (100.0%) | 2 (40.0%) |  |
| EASI (M ± SD) | 2.59 ± 2.70 | 4.00 ± 4.00 | 189.0 | 0.307 |  | 38.20 | 19.90 ± 6.60 |  |
| Visible lesion | 8 (61.5%) | 27 (79.4%) | 1.580 | 0.209 |  | 1 (100.0%) | 4 (80.0%) |  |
| Disease duration (years; M ± SD) | 28.5 ± 14.0 | 28.9 ± 16.4 | 213.0 | 0849 |  | 17.0 | 15.2 ± 20.9 |  |
| DLQI | 6.92 ± 8.41 | 5.17 ± 5.15 | 222.0 | 0.785 |  | 24.00 | 11.60 ± 8.32 |  |
| ***PSO subsample*** |  |  |  |  |  | *n = 9* |  |  |
| n (%) | 24 (27.6) | 63 (72.4) |  |  |  | 2 (22.2%) | 7 (77.8%) |  |
| Age (years; M ± SD) | 41.3 ± 18.3 | 50.9 ± 12.7 | 515.5 | *0.022* |  | 35.5 ± 2.1 | 48.6 ± 13.5 |  |
| Women | 15 (62.5%) | 19 (30.2%) | 7.635 | *0.006* |  | 1 (50.0%) | 4 (57.1%) |  |
| PASI (M ± SD) | 2.02 ± 2.56 | 1.75 ± 2.63 | 661.0 | 0.355 |  | 27.15 ± 4.17 | 15.21 ± 9.36 |  |
| Visible lesion | 13 (54.2%) | 32 (50.8%) | 0.079 | 0.778 |  | 2 (100.0%) | 7 (100.0%) |  |
| Disease duration (years; M ± SD) | 19.1 ± 15.4 | 21.1 ± 16.8 | 722.0 | 0.747 |  | 28.0 ± 5.2 | 20.7 ± 19.4 |  |
| DLQI | 4.92 ± 5.36 | 3.37 ± 5.73 | 546.5 | *0.042* |  | 7.00 ± 2.74 | 9.00 ± 3.83 |  |

DLQI, dermatology life quality index; EASI, eczema area severity index; M, mean; PASI, psoriasis area severity index; SD standard deviation.

**TABLE S3** Comparison of age, sex, disease severity and duration, and quality of life in patients with chronic inflammatory skin diseases with and without social anxiety disorder (SAD; lifetime), stratified according to disease severity (mild on the left side; moderate-to-severe on the right side).

|  | **Severity: Mild (n = 136)** | | | | **Severity: Moderte-to-severe (n = 15)** | |
| --- | --- | --- | --- | --- | --- | --- |
| ***Total study sample*** | ***SAD +*** | ***SAD –*** | ***Statistics*** |  | ***SAD +*** | ***SAD –*** |
|  |  |  | *U/ χ^2^* | *p* |  |  |
| n (%) | 22 (16.2) | 114 (83.8) |  |  | 4 (33.3) | 11 (73.3) |
| Age (years; M ± SD) | 35.7 ± 14.2 | 47.1 ± 15.8 | 745.0 | *0.003* | 36.5 ± 15.2 | 47.7 ± 18.6 |
| Women | 14 (63.6%) | 43 (37.7%) | 5.088 | *0.024* | 3 (75.0%) | 5 (45.5%) |
| Visible lesion | 13 (59.1%) | 67 (59.8%) | 0.004 | 0.949 | 4 (100.0%) | 10 (90.9%) |
| Disease duration (years; M ± SD) | 18.0 ± 14.9 | 24.5 ± 16.7 | 950.0 | 0.090 | 13.0 ± 12.2 | 21.4 ± 19.8 |
| DLQI | 5.64 ± 6.64 | 4.22 ± 5.71 | 1034.5 | 0.188 | 15.50 ± 8.27 | 9.00 ± 5.31 |
| ***AD subsample*** |  |  |  |  | *n = 6* |  |
| n (%) | 7 (14.3) | 42 (85.7) |  |  | 2 (33.3%) | 4 (66.7%) |
| Age (years; M ± SD) | 30.7 ± 12.7 | 41.3 ± 16.7 | 82.5 | 0.065 | 28.0 ± 12.7 | 51.0 ± 16.7 |
| Women | 4 (57.1%) | 19 (45.2%) | 0.341 | 0.559 | 1 (50.0%) | 2 (50.0%) |
| EASI (M ± SD) | 3.46 ± 3.32 | 3.66 ± 3.82 | 143.0 | 0.909 | 27.35 ± 15.34 | 20.75 ± 7.30 |
| Visible lesion | 6 (85.7%) | 29 (72.5%) | 0.547 | 0.459 | 2 (100.0%) | 3 (75.0%) |
| Disease duration (years; M ± SD) | 28.0 ± 14.5 | 29.0 ± 16.0 | 130.0 | 0.765 | 8.5 ± 12.0 | 19.0 ± 22.0 |
| DLQI | 10.00 ± 9.73 | 4.90 ± 5.14 | 102.0 | 0.208 | 22.50 ± 2.12 | 9.25 ± 7.46 |
| ***PSO subsample*** |  |  |  |  | *n = 9* |  |
| n (%) | 15 (17.2) | 72 (82.8) |  |  | 2 (22.2%) | 7 (77.8%) |
| Age (years; M ± SD) | 38.1 ± 14.6 | 50.4 ± 14.3 | 299.0 | *0.007* | 45.0 ± 15.6 | 45.9 ± 20.7 |
| Women | 10 (66.7%) | 24 (33.3%) | 5.794 | *0.016* | 2 (100.0%) | 3 (42.9%) |
| PASI (M ± SD) | 1.45 ± 2.90 | 1.90 ± 2.54 | 435.0 | 0.226 | 18.70 ± 7.78 | 17.63 ± 10.82 |
| Visible lesion | 7 (46.7%) | 38 (52.8%) | 0.186 | 0.667 | 2 (100.0%) | 7 (100.0%) |
| Disease duration (years; M ± SD) | 13.3 ± 13.1 | 22.1 ± 16.7 | 374.0 | 0.062 | 17.5 ± 14.8 | 23.0 ± 20.2 |
| DLQI | 3.60 ± 3.42 | 3.83 ± 6.02 | 447.5 | 0.287 | 8.50 ± 2.12 | 8.83 ± 4.17 |

*Abbr.:* DLQI, dermatology life quality index; EASI, eczema area severity index; M, mean; PASI, psoriasis area severity index; SD, standard deviation

**TABLE S4** Comparison of lifetime prevalence of shame-related disorders (SRD), body dysmorphic disorder (BDD), and social anxiety disorder (SAD), among patients treated with biologics vs. no biologics (left side) as well as patients treated with biologics compared to those treated with topicals only (right side).

| **Total study sample** | **Biologics +** | **Biologics –** | **Statistics** |  |  | **Biologics +** | **Topicals only** | **Statistics** |  |
| --- | --- | --- | --- | --- | --- | --- | --- | --- | --- |
|  |  |  | ***U/ χ^2^*** | ***p*** |  |  |  | ***U/ χ^2^*** | ***p*** |
| n (%) | 91 (60.3) | 60 (39.7) |  |  |  | 91 (79.1) | 24 (20.9) |  |  |
| SRD | 33 (36.3) | 15 (25.0) | 2.116 | 0.146 |  | 33 (36.3) | 5 (20.8) | 2.044 | 0.153 |
| BDD | 29 (31.9) | 11 (18.3) | 3.402 | 0.065 |  | 29 (31.9) | 3 (12.5) | 3.547 | 0.060 |
| SAD | 17 (18.7) | 9 (15.0) | 0.344 | 0.558 |  | 17 (18.7) | 3 (12.5) | 0.505 | 0.477 |
| ***AD subsample*** | ***35 (63.6)*** | ***20 (36.4)*** |  |  |  | ***35 (77.8)*** | ***10 (22.2)*** |  |  |
| SRD | 11 (31.4) | 5 (25.0) | 0.255 | 0.614 |  | 11 (31.4) | 2 (20.0) | 0.495 | 0.482 |
| BDD | 10 (28.6) | 4 (20.0) | 0.493 | 0.483 |  | 10 (28.6) | 2 (20.0) | 0.292 | 0.589 |
| SAD | 5 (14.3) | 4 (20.0) | 0.304 | 0.582 |  | 5 (14.3) | 1 (10.0) | 0.124 | 0.725 |
| ***PSO subsample*** | ***56 (58.3)*** | ***40 (41.7)*** |  |  |  | ***56 (80.0)*** | ***14 (20.0)*** |  |  |
| SRD | 22 (39.3) | 10 (25.0) | 2.143 | 0.143 |  | 22 (39.3) | 3 (21.4) | 1.556 | 0.212 |
| BDD | 19 (33.9) | 7 (17.5) | 3.189 | 0.074 |  | 19 (33.9) | 1 (7.1) | 3.938 | 0.054 |
| SAD | 12 (21.4) | 5 (12.5) | 1.276 | 0.259 |  | 12 (21.4) | 2 (14.3) | 0.357 | 0.550 |

*Abbr.:* SRD, shame-related disorders; BDD, body dysmorphic disorder; SAD, social anxiety disorder
